# Osteoprotective Effect of Cordycepin on Estrogen Deficiency-Induced Osteoporosis *In Vitro* and *In Vivo*

**DOI:** 10.1155/2015/423869

**Published:** 2015-03-22

**Authors:** Da-wei Zhang, Hualiang Deng, Wei Qi, Guang-yue Zhao, Xiao-rui Cao

**Affiliations:** ^1^Department of Orthopedics, Xi Jing Hospital, The Fourth Military Medical University, Xi'an 710032, China; ^2^Shandong University of Traditional Chinese Medicine, Jinan 250355, China; ^3^The Surgery Department of 520th Hospital of PLA, Mianyang 621000, China

## Abstract

The purpose of this study was to verify the effect of cordycepin on ovariectomized osteopenic rats. Fifty Wistar female rats used were divided into 5 groups: (1) sham-operation rats (control), (2) ovariectomized (OVX) rats with osteopenia, and (3) OVX'd rats with osteopenia treated with cordycepin (5 mg, 10 mg, and 20 mg) for 8 weeks. After the rats were treated orally with cordycepin, serum alkaline phosphatase (ALP), tartrate resistant acid phosphatase (TRAP), serum osteocalcin (OC), homocysteine (HCY) , C-terminal crosslinked telopeptides of collagen type I (CTX) level, and oxidative stress were examined, respectively. The femoral neck was used for mechanical compression testing. At the same time, we further investigated the effect of cordycepin *in vitro* assay. The beneficial effects of cordycepin on improvement of osteoporosis in rats were attributable mainly to decrease ALP activity, TRAP activity, and CTX level. At the same time, cordycepin also increases the OC level in ovariectomized osteopenic rats. The histological examination clearly showed that dietary cordycepin can prevent bone loss caused by estrogen deficiency. These experimental results suggest that complement cordycepin is protective after ovariectomized osteopenic in specific way.

## 1. Introduction

Osteoporosis is a major concern in public health care and the disease has severe consequences if untreated [1, 2]. It is characterized by low bone mineral density (BMD) and loss of the structural and biomechanical properties that are required to maintain bone homeostasis. Bone is a metabolically active tissue that undergoes remodeling throughout life, with roughly 5% remodeled at any time [3]. Over several weeks, a bone remodeling unit (BMU) will develop that incorporates several cell types, including osteoclasts, osteoblasts, and osteocytes. The loss of sclerostin and alterations in other secreted cytokines and chemotactic factors promote BMU's formation. Despite current treatment options that include vitamin D, hormone, and bisphosphonates therapy, osteoporosis results in significant morbidity and mortality. Development of novel therapies is vital for therapy against osteolytic bone diseases.

The herbal kingdom is a wide field to search for natural effective osteoporosis protective agent that has no side effects. As potential alternative treatments for osteoporosis, the preventive and therapeutic effects of natural products derived from plants have been reported [4–6].* Cordyceps* is a genus of the family Clavicipitaceae that has been used in traditional Oriental medicine for centuries. Recent studies have demonstrated that the bioactive components isolated from this genus have various pharmacological actions [7–9]. Among them, cordycepin, also known as 3-deoxyadenosine, has been shown to possess multiple pharmacological activities such as inhibition of tumour growth, modulation of the immune response, and suppression of reactive oxygen species [10]. In the former paper, we have reported that cordycepin can act as anti-inflammatory agent in magnesium silicate-induced inflammation in osteoporosis [11]. However, the role of cordycepin in estrogen deficiency-induced osteoporosis in ovariectomized rats has not been investigated. The aim of the present investigation was to discover cordycepin for effective osteoporosis treatment* in vivo* and* in vitro*.

## 2. Materials and Methods

### 2.1. Animals

Wistar rats (weighing 225 ± 25 g) were used in the study. This study was performed in accordance with the Guide for the Care and Use of Laboratory Animals. Care was taken to minimize discomfort, distress, and pain to the animals. The study was submitted to, and approved by, the Fourth Military Medical University institutional ethics committee.

### 2.2. Drugs

Cordycepin with 98% purification was obtained following the extraction and separation using a column chromatographic method [12].

### 2.3. Experimental Design

Fifty rats were randomly divided into five groups of animals, four ovariectomized (OVX) and another given a sham-operation (control). Then groups 1 (sham) and 2 (OVX) were treated orally with 10 mL of saline; group 3, group 4, and group 5 were treated orally with cordycepin (5 mg, 10 mg, and 20 mg) for 8 weeks, respectively. Cordycepin was dissolved in distilled water and administrated orally twice daily using a feeding needle for 21 days, and control group received double distilled water instead of cordycepin. Body weight of the animals was recorded weekly.

On the last day of treatment and necropsy, blood was collected from dorsal aorta under ether anesthesia. After centrifugation, serum was harvested and kept at −20°C until analysis. The femoral neck was processed for mechanical testing. The entire fifth lumbar vertebrae and one tibia were processed for histology.

### 2.4. Mechanical Testing

The mechanical strength of the femoral neck was measured by applying a vertical load to the femoral head using a Shimadzu EZ-1 pressure system. The fracture load was recorded at the peak force as Newton (N) at the point that the femoral neck fractured [13].

### 2.5. Histomorphometry of Osteoblast Surface

The tibia and the lumbar vertebrae were decalcified in formic acid, embedded in paraffin, and longitudinally sectioned. Histomorphometric analyses were made by tracing the section image onto a digitizing platen with the aid of a camera lucida attachment on the microscope and Osteomeasure bone analysis software. To reveal osteoclasts, sections were stained for immunoreactivity to cathepsin K, an osteoclast marker [14]. Osteoblast perimeter was determined by scoring osteoblasts in direct contact with cancellous bone surfaces.

### 2.6. Plasma Enzyme Measurements

ALP and TRAP activity were determined by nitrophenol based method as described by Bessy et al. [15] and Godkar [16], respectively.

### 2.7. Plasma Proteins Measurements

Serum osteocalcin (OC) content was determined using an Osteocalcin EIA kit (Xinyubio-Technology, Inc., China) as described in the manufacturer's directions. Homocysteine (HCY) was measured by use of an enzymatic fluorescence polarization immunoassay on an Axsym analyzer (Abbott, Wiesbaden, Germany). C-terminal crosslinked telopeptides of collagen type I (CTX) were quantified by ELISA (Sunbio, Inc., China).

### 2.8. *In Vitro* Assay and Alkaline Phosphatase (ALP) and Tartrate Resistant Acid Phosphatase (TRAP)

The murine mesenchymal stem cell line was purchased from the Beijing Lihao Inc., China, and grown in a DMEM medium supplemented with 10% fetal bovine serum (FBS), penicillin (100 U/mL), and streptomycin (100 *μ*g/mL). All cultured cells were incubated in a humidified atmosphere at 37°C and at 5% CO_2_. The study used cells with passages 5–10 (after purchase) for all experiments in cell lines. Cells (3 × 10^3^ cells/well) were incubated in a 96-well plate overnight and cotreated with different concentrations of cordycepin in the medium for 48 h. ALP activity was measured in total cell lysates after homogenization in a buffer containing 1 mmol/L Tris–HCl (pH 8.8), 0.5% Triton X-100, 10 mmol/L Mg2+, and 5 mmol/L p-nitrophenylphosphate as substrates. The absorbance was read at 405 nm. The differentiated osteoclast cells from monocytes were measured by a TRAP activity assay and staining using the Acid-Phosphatase Kit (Shanghai Jinma Biological Technology, Inc., China).

### 2.9. Oxidative Stress Assay

In serum, glutathione peroxidase (GPx) activity, glutathione reductase (GR) activity, catalase (CAT) activity, Na^+^K^+^ATPase activity, and glutathione S transferase (GST) activity were quantified by ELISA (Sunbio, Inc., China).

### 2.10. Statistical Analysis

Data were expressed as the mean ± S.E.M. and the results analyzed by ANOVA followed by Dunnett's *t*-test. A *P* value of <0.05 was considered significant.

## 3. Results and Discussion

**Figure 1:**
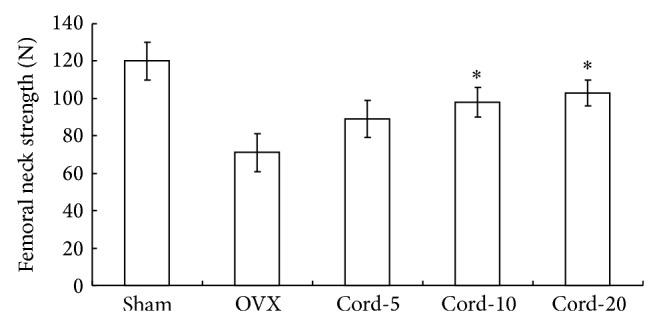
Effects of cordycepin on mechanical strength of the femoral neck. The data are presented as mean ± SD (*n* = 10 per group). ^*^ *P* < 0.05 as compared with corresponding values in saline-treated OVX. Bone strength of the femoral neck was significantly lower in the saline-treated OVX compared with the sham group and cordycepin treatment group.

Ovariectomized (OVX) animal models, in a variety of species, have been used to evaluate the mechanism of or to assess the effect of drugs on osteoporosis. Mechanical strength of bones is the most important parameter related to fracture risk. Therefore, this study first investigated the effect of cordycepin on mechanical strength in OVX osteopenic rats. The average maximum fracture loading to the femoral necks was lower in the OVX group compared with the sham group (Figure 1). Mechanical strength was significantly increased by treatment with cordycepin. It indicates that cordycepin had the positive effect on ovariectomized osteopenic rats.

**Figure 2:**
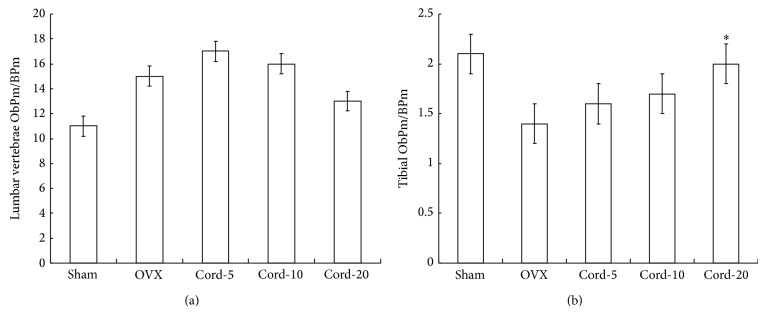
Effects of cordycepin on bone ObPm/BPm. Data are expressed as the mean ± SEM (*n* = 10 per group). ^*^ *P* < 0.05, significant difference from vehicle-treated OVX rats.

Based on the results of mechanical strength, the trabecular number and thickness were studied. In the current study, we found that treatment of osteopenic OVX rats with cordycepin significantly increased maximal load compared to OVX animals. At the end of the 8-week treatment period, osteoblast surface in the lumbar vertebrae was not affected by OVX or any treatment (Figure 2(a)). A different cellular response was observed in the proximal tibial metaphysis. Cordycepin treatment caused a 3-fold increase in osteoblast surface compared with that in OVX rats (*P* < 0.05) (Figure 2(b)). It indicates that cordycepin administration improved bone strength mainly by increasing trabecular thickness. It agrees with the report of Ulrich [17].

**Figure 3:**
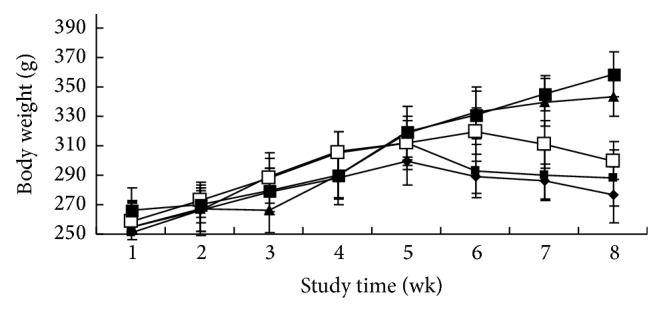
Effects of cordycepin on body weight changes for the study (◆sham group, ■OVX group, □cordycepin-5 group, ▲cordycepin-20 group, and ●cordycepin-10 group). Body weights were higher in the ovariectomized (OVX) animals than in sham-operated ones. Cordycepin-20 had similar body weights with the OVX animals in the former 5 weeks. From 5 weeks after the treatment was initiated, cordycepin-20 significantly increased body weights compared to sham group.

Estrogen deficiency induces increased body weight in ovariectomized rats. The body weight gain pattern is shown in Figure 3. By the end of the fifth week, the ovariectomized rats gained significant weight compared to all other groups. From 5 weeks after the treatment was initiated, cordycepin-20 significantly increased body weights compared to sham group (Figure 3). The increase in rats' body weight in the cordycepin groups in the present study could be due to increased food intake as a result of lower leptin secretion though the impact is less severe compared to OVX rats [18].

**Figure 4:**
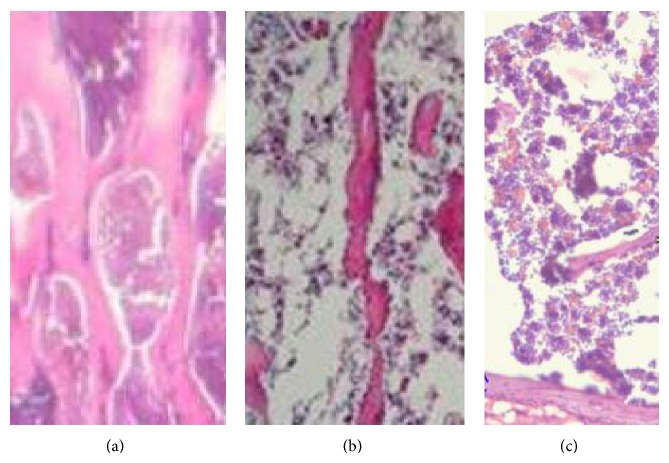
Histology of lumbar vertebrae. The bone structure was photographed under a light microscope. It shows that there was a significant trabecular bone loss in the OVX rat (b), whereas the cordycepin-20 treatment rat section (c) seems near normal compared with sham-operated animals (a).

Bone histomorphometry was also performed to determine the effects of cordycepin treatment on cancellous bone mass and levels of bone formation and resorption. A sample photomicrograph is presented in Figure 4. It is quite clear that trabecular bone loss is much higher in the vertebrae of rat with OVX (Figure 4(b)), whereas the vertebrae of cordycepin-fed OVX rat (Figure 4(a)) appear to be near normal (Figure 4(c)).

The current studies demonstrated that systemic treatment with cordycepin has a strong bone anabolic effect in OVX rats. The mechanism of it also needs to be studied in this paper.

**Figure 5:**
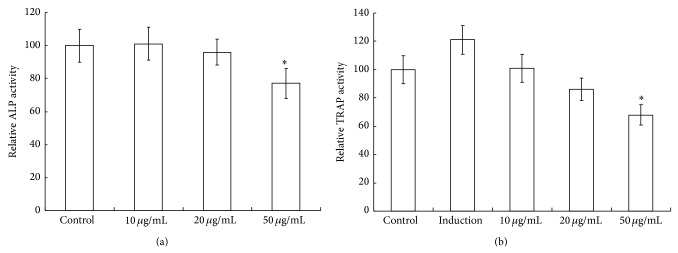
*In vitro* assay cordycepin on ALP activity and TRAP activity. Treatment of 50 *μ*g/mL of cordycepin showed significantly decreased ALP activity and TRAP activity. Values are mean ± SEM. *n* = 10. ^*^ *P* < 0.05 compared to the control group at the same timepoint.

ALP is a noncollagenous protein secreted by osteoblast, which is essential for bone mineralization [19]. Increased ALP level in serum has been observed in conditions such as rapid bone loss [20] and fracture risk [21, 22]. TRAP is secreted by osteoclasts during bone resorption, and serum TRAP activity correlates with resorptive activity in disorders of bone metabolism. In the present study significant increase in ALP and TRAP levels was observed in OVX control (Table 1). On the contrary, cordycepin significantly decreased ALP and TRAP levels (*P* < 0.01). Based on the above results, we further investigated the effect of cordycepin* in vitro* assay. Treatment of 50 *μ*g/mL of cordycepin showed significantly decreased ALP activity (Figure 5(a)) and TRAP activity (Figure 5(b)). It suggested that the potency of cordycepin is due to decrease ALP activity, TRAP activity in OVX rats.

Osteocalcin (OC), homocysteine (HCY), and collagen type I (CTX) are known as serum markers reflecting osteoblast activities including bone formation and turnover [23–25]. The effects of treatment with cordycepin on OC, HCY, and CTX level were shown in Table 2. Treatment of 50 *μ*g/mL of cordycepin increased OC level (*P* < 0.01). It also significantly decreased CTX level (*P* < 0.01). However, compared with OVX control, there were no significant differences in the increase of HCY content in cordycepin groups (Table 2). These results suggested that the treatment with cordycepin induces the secretion of OC as well as decreased secretion of CTX after oral administration.

Oxidative stress and free radicals have been implicated in the pathogenesis of osteoporosis. Therefore, antioxidant compounds have the potential to be used in the prevention and treatment of the disease. Reduced glutathione (GSH) is one of the primary endogenous antioxidant defense systems, which removes hydrogen peroxide and lipid peroxides. Decline in GSH levels could either increase or reflect oxidative status [26, 27]. Therefore, the measurement of endogenous antioxidants enzymes, that is, GPx, GR, CAT, and GST, as well as Na^+^K^+^ATPase, has been performed to estimate the amount of oxidative stress. Activities of various antioxidant enzymes and Na^+^K^+^ATPase of different groups have been listed in Table 3. The activity of endogenous antioxidant enzymes was decreased significantly (*P* < 0.01) in the OVX group, as compared to the sham group, whereas in the cordycepin group, cordycepin treatment showed a significant (*P* < 0.05–0.01) restoration in the level of various enzyme as compared with OVX group.

In conclusion, our findings first showed that oral administration of cordycepin can counteract the bone loss in an experimental model of established osteoporosis. These findings suggested that the mechanism of cordycepin is due to decrease ALP activity and TRAP activity both* in vitro* and* in vivo.* At the same time, oral administration of cordycepin can increase the OC level and decrease CTX and CTX level as well as restoring the oxidative stress in OVX animals. This suggests that cordycepin may be a good natural herbal medicine candidate for the treatment of osteoporosis.

## Figures and Tables

**Table 1:** Effects of cordycepin on plasma enzymes in ovariectomized rats.

Groups	TRAP level (uM)	ALP level (mM)
Sham	0.22 ± 0.11^**^	3.25 ± 0.12^**^
OVX	0.82 ± 0.11	7.21 ± 0.10
Cordycepin-20	0.33 ± 0.02^**^	4.11 ± 0.04^*^
Cordycepin-10	0.61 ± 0.02	4.82 ± 0.06^*^
Cordycepin-5	0.70 ± 0.03	7.22 ± 0.01

Values are mean ± SEM. *n* = 10. ^*^ *P* < 0.05 versus OVX control; ^**^ *P* < 0.01 versus OVX control.

**Table 2:** Effects of cordycepin on plasma proteins.

Groups	Serum OC (ng/mL)	Serum HCY (*μ*mol/L)	Serum CTX (ng/mL)
Sham	81.0 ± 5.0	7.7 ± 1.1	75.5 ± 4.2
OVX	61.4 ± 5.1	9.2 ± 2.0	100.3 ± 5.0
Cordycepin-20	84.1 ± 5.1^**^	8.0 ± 1.0	79.0 ± 8.1^**^
Cordycepin-10	73.6 ± 4.0^*^	9.2 ± 3.3	85.1 ± 9.0
Cordycepin-5	70.6 ± 3.2	8.0 ± 2.1	90.3 ± 4.4

Values are mean ± SEM. *n* = 10. ^*^ *P* < 0.05 versus OVX control; ^**^ *P* < 0.01 versus OVX control.

**Table 3:** Effect of cordycepin on the activity of various enzymes.

Different groups	GPx	GR	GST	CAT	Na^+^K^+^ATPase
Sham	15.98 ± 1.23^***^	35.55 ± 2.51^***^	17.00 ± 1.22^**^	7.11 ± 0.33^*^	4.52 ± 0.32^**^
OVX	8.01 ± 0.42	20.88 ± 2.11	10.07 ± 1.11	4.22 ± 0.13	2.00 ± 0.13
Cordycepin-20	13.16 ± 1.32^**^	29.01 ± 2.21^***^	13.66 ± 0.90^*^	5.78 ± 0.20^*^	4.11 ± 0.22^*^
Cordycepin-10	9.10 ± 1.02	26.00 ± 2.22	13.00 ± 0.91	4.70 ± 0.21	3.51 ± 0.23
Cordycepin-5	8.16 ± 1.31	21.21 ± 2.20	10.10 ± 0.92	4.28 ± 0.33^*^	2.10 ± 0.11

Values are shown as means ± SEM. ^*^ *P* < 0.05 versus OVX group, ^**^ *P* < 0.01 versus OVX group, and ^***^ *P* < 0.001 versus OVX group.
